# What do patients care most about in China’s public hospitals? Interviews with patients in Jiangsu Province

**DOI:** 10.1186/s12913-018-2903-6

**Published:** 2018-02-08

**Authors:** Xuanxuan Wang, Rongqin Jiang, Jingxian Li, Jiaying Chen, Bo Burström, Kristina Burström

**Affiliations:** 1 School of Health Policy and Management, Nanjing Medical University, Nanjing, China; 2 Health Outcomes and Economic Evaluation Research Group, Stockholm Centre for Healthcare Ethics, Department of Learning, Informatics, Management and Ethics, Karolinska Institutet, Stockholm, Sweden; 3 Centre for Health Policy Studies, Nanjing Medical University, Nanjing, China; 4 Creative Health Policy Research Group, Nanjing Medical University, Nanjing, China; 5 Equity and Health Policy Research Group, Department of Public Health Sciences, Karolinska Institutet, Stockholm, Sweden

**Keywords:** China’s public hospital reform, Evaluation, Interviews, Patient-reported experience measures

## Abstract

**Background:**

Evaluations on different aspects of the performance of public hospitals in China have been conducted, usually based on indicators developed by literature review and expert suggestions. The patient perspective was not always considered. This study aims to identify what patients care most about in China’s public hospitals exclusively from a patient perspective.

**Methods:**

A mix of stratified sampling and typical sampling was used to select 15 public hospitals in Jiangsu Province of China. In each sampled hospital, a convenient sample of six outpatients and six inpatients was selected to conduct face-to-face individual interviews. An interview guide consisting of six open-ended questions was designed. Donabedian’s quality of care framework was applied to categorize themes and subthemes, which were generated from patients’ interviews by using the conventional content analysis approach. Frequencies of themes and subthemes were counted.

**Results:**

Nine key themes were identified regarding patients’ concerns about hospital care, which were *environment and facilities*, *professional competence*, *hospital reputation*, and *morals of medical staff* in the “structure” category of Donabedian’s framework, *caring attitudes and emotional support*, *medical costs*, *communication and information*, and *efficiency and coordination of care* in the “process” category, and *health outcomes* in the “outcome” category.

**Conclusions:**

This study has identified and prioritized the aspects that patients care most about in China’s public hospitals in Jiangsu Province exclusively from a patient perspective. A measurement tool of patient-reported experiences in public hospitals could be built based on this study. Efforts should be made to represent the patient perspective to further improve the reform of public hospitals in China.

**Electronic supplementary material:**

The online version of this article (10.1186/s12913-018-2903-6) contains supplementary material, which is available to authorized users.

## Background

As the backbone of health services in China, public hospitals provide 89% of hospital bed-days and 92% of outpatient visits [1, 2]. Patients in China have low trust in primary care facilities and there are no formal referral systems [3]. As a result, patients often seek care at public hospitals even for minor health problems, which loads heavy burden of outpatient care on physicians at large public hospitals in addition to their routine specialist practice [4]. The majority of Chinese public hospitals are both regulated and operated by the government [5]. Government financing, charges on health services, and 15% mark-up on drugs were the main sources of revenues for Chinese public hospitals in the past [4]. But due to insufficient financing and distortedly low prices on medical services, hospital directors and physicians have been encouraged to overprescribe drugs and tests to make profits [3]. In 2009, the Chinese Government launched a nationwide health-care reform, and identified the public hospital reform as one core target [6]. The desired results of China’s health-care reform cannot be fully achieved unless the efficiency in public hospitals has been improved and the growth of health expenditure has been under control [3, 7].

Since the initiation of the public hospital reform, evaluations on different aspects of the performance of public hospitals have been conducted in the past several years, regarding hospital governance [8], hospital efficiency [9], clinical quality [10], health expenditure and utilization [11], and the doctor-patient relationship from physicians’ perspectives [12].

It has long been recognized internationally that patients’ perspectives on health and health care are important to improve the quality of health care [13]. As to measures of patients’ perspectives, there are two main categories. One is Patient-Reported Outcome Measures (PROMs), which record patients’ own assessment of their health and health-related quality of life [14]. The other is Patient-Reported Experience Measures (PREMs), which capture patients’ evaluation of their satisfaction with, or experience of, health care services [15].

However, when assessing hospital care, indicators used are usually developed by literature review and expert suggestions rather than patients’ experiences of hospital care [16]. It is often the case that patients’ priorities are omitted and questions are fashioned by the medical staff’s perceptions and definitions of quality of hospital care [17]. Robust evidence has demonstrated that if designed and administered appropriately, measures on patients’ experiences are capable of capturing unique and important dimensions of quality of care [18]. Patients’ experiences could provide insights into areas that need improvement, provoke potential solutions, and facilitate changes [19, 20].

Efforts have been made in both high and low-income settings to develop measurement tools on patients’ experiences of hospital care such as the Hospital Consumer Assessment of Health Care Providers and Systems in US [21], the Picker Patient Experience Questionnaire in some European countries [22], the Patient Experiences Questionnaire in Norway [23], and the questionnaires designed to assess patients perceptions of health care quality in Ethiopia and India [24, 25].

Nevertheless, in China patients’ perspectives are seldom considered when assessing hospital care. In a previous study, patient satisfaction on health care services in hospitals was measured, but only the composite score was reported [26]. In two other studies concerning patient satisfaction in public hospitals, one focused on patients’ perceptions of hospital’s social responsibility [27] and the other only included outpatients [28].

Since quality of care is difficult to quantify or define [29], in order to elicit patients’ views on which aspects of hospital care they care most about, a qualitative study design using individual interviews and open-ended questions may be useful. By conducting individual interviews using open-ended questions, an in-depth understanding of what patients are most concerned with when seeking health services in hospitals could be uncovered [30, 31]. Apart from avoiding directing or biasing initial responses [32], using open-ended questions could enable participants to tell their stories on a more personal level and gain confidence in conveying their views [33]. In addition, analysis of patient interviews could form the basis for creating a scale to measure patients’ experiences of health care [34].

Due to the complexity of patients’ views on hospital care, Donabedian’s framework of quality of care was used in this study to present the themes and subthemes elicited from patients’ interviews [35]. According to Donabedian’s framework, the information about the quality of care can be classified under three categories: “structure,” “process,” and “outcome.” “Structure” denotes the attributes of the settings in which care occurs, including material resources, human resources, and organizational structure. “Process” denotes what is actually done in giving and receiving care and includes patient’s activities in seeking care and carrying it out as well as the practitioner’s activities in making a diagnosis and recommending or implementing treatment. “Outcome” denotes the effects of care on the health status of patients and populations [35, 36].

This study aims to identify what patients care most about in China’s public hospitals by conducting individual interviews with both outpatients and inpatients.

## Methods

### Setting

The sampled public hospitals in this study were all located at Jiangsu Province, as Jiangsu is one of the four pilot provinces to implement a comprehensive health-care reform, and Zhenjiang City in Jiangsu Provinces is one of the 17 first-batch pilot cities that implemented pilot public hospital reforms.

A mix of stratified sampling and typical sampling was used in this study to select sampled public hospitals. In the first step, five cities in the northern, middle and southern parts of Jiangsu Provinces were selected to reflect the different economic levels of the province. To illustrate, Zhenjiang and Changzhou in the south, Yangzhou in the middle, and Liangyungang and Yancheng in the north were selected, as their economic development was at the middle level of each region. In the second step, two counties in each sampled city were selected. Of the two sampled counties, one was in the progress of public hospital reform, while the other had no breakthroughs yet. The two counties had similar geographic, environment and demographic characteristics and similar level of health resources. In the third step, the biggest municipal public hospital in each sampled city and the biggest county general hospital in each sampled county were selected as sampled hospitals. Altogether, 15 public hospitals were sampled in this study.

### Participants

In each sampled hospital, a convenient sample of six outpatients and six inpatients was selected to conduct face-to-face individual interviews. Required number and sampling criteria of patients in each sampled hospital were presented in Table 1. A total of 180 patients participated in the interviews carried out in August, 2015.

**Table 1:** Required number and sampling criteria of patients in each sampled hospital

Type of patients	Departments of internal medicine		Departments of surgery	
	Urban residents	Rural residents	Urban residents	Rural residents
Outpatients	2	1	2	1
Inpatients	1	2	1	2
Total	3	3	3	3

### Training of interviewers

Ten interviewers conducted interviews, with two forming a group responsible for all the interviews in each sampled city. In each group, one interviewer was either a teacher of Nanjing Medical University or a member of the project research group, and the other was a master student in the School of Health Policy and Management of Nanjing Medical University.

Before the survey, a workshop was held to train interviewers on the goals and objectives of the study, the interview guide and central questions (Additional file 1), and interpersonal interview techniques. Role plays were conducted at the workshop and pilot interviews were undertaken afterwards to ensure consistency in the understanding of the interview guide and proficiency in the application of interview techniques. Having passed the assessment by the principal investigator, all the ten interviewers were allowed to take part in the survey.

### Data collection

To elicit a wide range of views and experiences, an interview guide mainly consisting of six central open-ended questions was designed (Additional file 1). Prompts and follow up questions were used during the interview to further solicit patient’s views and experiences. The interview guide was designed to elicit patients’ experiences without setting predetermined criteria in the range of care or answering options.

Each interview lasted 30-40 min, recorded with a digital recorder and transcribed verbatim. Notes were also taken during the interview. The transcribed text was compared with the recording and adjusted if necessary.

### Ethical consideration

According to *The Ethical Review Policy of Human Biomedical Research* issued by China’s former Ministry of Health in 2007, ethics approval did not have to be applied for this study where only individual interviews were conducted [37]. As stated in the national policy, research on human physiological and pathological phenomena and disease diagnosis, treatment and prevention through modern physical, chemical and biological approach on human bodies and experimental applications on human bodies through medical technologies or products produced by biomedical research should apply for ethics approval beforehand [37]. Although no official ethics approval should be sought for this study, the research group did follow standard procedures to obtain oral informed consent before each interview initiated. Having found a potential interviewee according to the sampling criteria, the interviewer introduced his/her own identity and the purpose of the interview. The interviewer then explained that this study was funded by the National Natural Science Foundation of China, which means that the hospital where the interview would be conducted had no influence on the study. The potential interviewee was also informed that his/her participation was voluntary and anonymous and no private information would be asked during the interview, including name, phone number, and home address. The interviewer also illustrated that the potential interviewee had the right to refuse to participate in the interview and the right to terminate participation at any time during the interview. The potential interviewee was also informed the usage of a digital recorder after the interview was initiated. If the potential interviewee authorized the interview, the interview would be conducted when the potential interviewee had completed his/her clinic visit without another third person on spot. Oral informed consent was obtained from each interviewee.

### Data analysis

To examine patients’ interviews, the approach of conventional content analysis was used [38]. A team of six researchers was built, with two working in a pair. Each pair was responsible for analyzing 60 transcripts of interviews. Three rounds of analyses were performed.

In the first round, all six researchers worked on the same five transcripts in order to achieve consistency in coding the text and produce a codebook of potential subthemes. At the beginning, they read the transcripts independently and obtained a sense of whole. Next, they highlighted words that contained key thoughts and concepts according to researchers’ own understanding. As the highlighting process continued, subthemes were generated, indicating the same key thoughts and concepts. During this highlighting process, two researchers in a pair held ongoing discussion and compared subthemes until an agreement was reached on the meaning and number of subthemes they generated from the transcripts. After that, a large group meeting was held with all six researchers to discuss the three sets of subthemes generated by three pairs. The group meeting ended with a consensus and consistency in coding the text and a codebook of subthemes was produced.

In the second round, researchers kept coding transcripts independently. Constant discussion went on about coding decisions and incorporating new subthemes into the codebook. Having completed coding transcripts, all six researchers merged and reduced subthemes, and categorized subthemes into key themes.

In the last round, the frequencies of themes and subthemes were counted so as to establish priorities in patients’ views and experiences of hospital care.

## Results

Of all the 180 interviewees, 139 had information on sex, among which 60% were male and 40% were female. There were 145 interviewees having information on age ranging from 21 to 91 years old, among which 37% were in the age group 21-44 years old, 35% fell into the 45-64 years old group, and the remaining 28% were 65 years old or above. The mean age was 51 years. Outpatients and inpatients accounted for 50%, respectively.

Nine key themes were identified in this study regarding the aspects that patients cared about most in public hospitals. These themes were organized according to Donabedian’s framework - “structure”, “process”, and “outcome” [35]. As shown in Table 2, the nine key themes were *environment and facilities*, *professional competence*, *hospital reputation*, *morals of medical staff*, *caring attitudes and emotional support*, *medical costs*, *communication and information*, *efficiency and coordination of care*, and *hospital outcomes*. Themes, subthemes and corresponding word frequencies were presented in Additional file 2.

**Table 2:** Key themes of patients’ concerns about hospital care

Structure	Process	Outcome
Environment and facilities	Caring attitudes and emotional support	Health outcomes
Professional competence	Medical costs	
Hospital reputation	Communication and information	
Morals of medical staff	Efficiency and coordination of care	

### “Structure” of patients’ concerns about hospital care

#### Theme one: Environment and facilities

Patients repeatedly commented on hospital environment and facilities in the interviews. A majority of patients stated that hospital hygiene and medical equipment were important to their perceptions of care in public hospitals. Patients commented on the distribution of clinical and non-clinical departments, the instruction of how to see a doctor, hospital canteen, parking availability, convenient facilities in the ward, and even the availability of up-to-date technology such as access to internet. Patients also mentioned their concerns about the easy access to public hospitals, including adjacency and developed public transportation to hospitals.

#### Theme two: Professional competence

The professional capacity of doctors in public hospitals was emphasized by patients in the interviews. Other highlighted aspects were doctors’ ability to prescribe proper medicines, fulfillment of responsibilities such as ward round and telephone follow-up by medical staff, and work experience of both doctors and nurses. Besides, some patients also expressed their concerns about the professional appearance presented by medical staff.

#### Theme three: Hospital reputation

Patients in this study were highly concerned with hospital scale and hospital reputation, especially the fame of medical competence of the hospital. Patients also stated that they cared about the rank of the hospital, i.e. whether it was a city-level comprehensive tertiary hospital. Some patients expressed their concerns about the drug supply of the hospital.

#### Theme four: Morals of medical staff

When speaking of medical staff, patients commented on the morals of medical professionals in the interviews. Patients expressed their worries that doctors might overprescribe medications and examinations. Some patients stressed their concerns about doctors’ medical ethics, e.g. acceptance of money and gifts from patients and provision of equal care to patients of different socioeconomic status.

### “Process” of patients’ concerns about hospital care

#### Theme five: Caring attitudes and emotional support

This theme was the one most frequently talked about among all the nine key themes identified. Patients stated that caring attitudes and emotional support from medical staff were important to their experience of care in public hospitals. A variety of aspects were stressed by patients ranging from the general attitude to more specific attributes such as sense of responsibility, politeness, patience, passion, tone, demonstration of caring and consolation, and building rapport with patients of both doctors and nurse. Respect for patients’ views and protection of patients’ privacy were also addressed by some participants.

#### Theme six: Medical costs

Individual interviews revealed that patients were much concerned about medical costs and reimbursement from health insurance. Patients commented on the costs of different types of health services including registration, drugs, medical examinations, surgeries, and nursing. Patients also highlighted the importance of health insurance reimbursement to them. Relevant discussion covered the ratio, scale and real-time reimbursement of health insurance. Some patients also talked about government financial support for serious diseases.

#### Theme seven: Communication and information

Patients expressed strong views on communication and information. They emphasized the provision of information on state of the illness, medications, medical examinations, treatment protocols, disease prognosis, medical costs and reimbursement policy. Good communication skills were also appreciated by patients such as using easy-to-understand language. A majority of patients also expressed their anticipation to be involved in decision making such as discussing their treatment protocols with doctors.

#### Theme eight: Efficiency and coordination of care

Patients commented on the waiting time of different services, including registration, diagnosis and treatment, getting medicines, taking medical examinations, paying fees and taking the elevator. Patients also expressed their expectations on effective coordination between multiple departments within the hospital such as the coordination across different organizations that enabled the patient to see a doctor or to be discharged from the hospital without delay.

### “Outcome” of patients’ concerns about hospital care

#### Theme nine: Health outcomes

Treatment effect was a common concern shared by patients about the care that they received in public hospitals. Patients in the interviews also addressed the length of recovery, pain control, unexpected outcomes, and disease diagnosis and prognosis.

## Discussion

### Public hospitals in China

Unlike many industrialized counties where only very ill patients are admitted to hospitals, patients in China can be admitted to public hospitals for chronic diseases and rehabilitation [2]. On top of inpatient care, Chinese public hospitals are overburdened with the load of outpatient care. As there are no formal referral systems, patients can go directly to public hospitals to seek outpatient care even for minor conditions [3]. The general public have low trust in the quality of care provided at primary care facilities; the demand for health services, consequently, increases and concentrates at large public hospitals [39]. Government financing is one of the primary sources of revenues for Chinese public hospitals. But due to insufficient financing, hospitals mainly survive on service fees [4]. Physicians’ salaries are also tied to hospital revenues in China. As a result, hospitals directors and physicians have been perversely incentivized to make up the gap of profits through over-prescriptions [40]. Findings in this present study have resonated with those prominent issues of Chinese public hospitals mentioned above from the patient perspective and identified the aspects of hospital care that patients are most concerned with.

### Aspects that patients care most about in public hospitals

Priorities of various aspects of hospital care have been determined in this study exclusively from the perspectives of patients rather than from the views of medical staff or health experts. To the best of our knowledge, this study is the first to explore what patients care about most in China’s public hospitals from a patient perspective by using individual interviews.

Of the nine key themes identified, four are concerned with the “structure” of hospital care, including *environment and facilities*, *professional competence*, *hospital reputation*, and *morals of medical staff*. Another four themes are classified into the “process” of hospital care, which are *caring attitudes and emotional support*, *medical costs*, *communication and information*, and *efficiency and coordination of care*. The remaining theme of *health outcomes* refers to the “outcome” of care in public hospitals.

For the theme of *professional competence*, patients in the interviews expressed their expectations on nurses and other medical staff apart from doctors. This finding indicates that patients are aware of and could distinguish the different roles of medical professionals in providing health care services within a hospital. Besides, patients’ emphasis on nurses’ professional capacity found in our study also underlines the overall shortage of nurses. Nursing shortage in China is more severe than that of some developed countries [41]. The particular low number of nurses makes the importance of this professional group even more salient.

Unlike previous findings, our study has disclosed a theme of *morals of medical staff*. Patients commented on medicines in the interviews from different angles. In terms of professional capacity, patients emphasized doctors’ ability to prescribe appropriate medicines. As regard to hospital reputation, patients commented on the drug supply of hospitals. When it comes to the moral issues, patients highlighted doctors’ prescribing behavior from the perspective of medical ethics rather than professionalism. These findings suggest that patients who are not trained or have medical expertise do have the ability to assess whether medical professionals deliver treatment appropriate to their health problems [42]. These results on the one hand reveal the moral issues in China’s public hospital, and on the other hand verify the importance of measuring patients’ experiences of care.

For the theme of *caring attitudes and emotional support*, the categories described by patients cover the general attitude to more specific attributes, and patients in our study did not differentiate much between doctors and nurses in their serving attitudes. All the attributes mentioned were expected to be possessed by both doctors and nurses. These findings are in consistency with a previous study in Australian patients [33]. Some interviewees also expressed their appreciation on respect and privacy protection. These results indicate that patients may seek a higher level of caring from medical staff than merely compassion and sympathy at public hospitals. Similar results have been observed in a qualitative study of Hong Kong patients [43].

For the theme of *medical costs*, patients in our study expressed their strong views on the costs of drugs, medical examinations, registration, surgeries, nursing and other costs. Although controlling health expenditure is a principal component of public hospital reform and a previous study has reported significant decrease in post-reform drug expenditures per visit [11], patients interviewed in our study were still highly concerned about their medical costs. Besides, patients also repeatedly mentioned health insurance reimbursement in the interviews from the reimbursed items covered by health insurance to the implementation of real-time reimbursement. In China there are three major social health insurance schemes, namely the Urban Employee Basic Medical Insurance (UEBMI), the Urban Resident Basic Medical Insurance (URBMI), and the New Rural Cooperative Medical Scheme (NCMS) [44]. Payroll taxes are the primary funding source for UEBMI premiums, and government subsidies are the major source for URBMI and NCMS premiums [44]. Each of the three insurance schemes has its own benefit package based on the scale of premium. UEBMI covers both inpatients and outpatient services, whereas URBMI and NCMS mainly cover inpatient services, with quite low proportion of outpatient services and selected chronic conditions reimbursed due to the small amount of premium [4]. Besides, these three health insurance schemes have different reimbursement caps. Patients with different types of insurance schemes, therefore, may have to pay different proportions of out-of-pocket money for the same health care with the same quality [44]. Due to variations in local government’s financial and management capacity and inequalities in benefit packages, although nearly universal insurance coverage has been accomplished in China since the initiation of the health-care reform, the benefits remain shallow [45]. Another key barrier to insurance use in China is the mobility of insurance management [4]. Currently, most patients have to go back to their registration city or county to get reimbursement, if they seek health care in other areas. Findings in this present study indicate that patients still suffer economic burden and barriers to insurance use when seeking care in public hospitals.

For the theme of *communication and information*, patients highlighted the importance of medical staff providing information on various aspects of care and possessing good communication skills. Similar findings have been shown in previous studies [46]. Participants in this study also emphasized their own involvement in decision making such as discussing treatment with doctors. This finding concurs with the results of other studies, suggesting that patients value their own engagement in hospital care and prefer individualized treatment achieved through collective decision making [47, 48].

For the theme of *efficiency and coordination of care*, relevant sections of interviews in this study were dominated by the topic of waiting time in public hospitals. Apart from time spent on waiting for seeing a doctor and taking the medical examinations, the waiting time for registration and paying fees were also commented on by patients, and even the waiting time for taking the elevator was mentioned. These phenomena might be probably caused by the fact that China has no strict referral system, and patients can go directly to public hospitals for all outpatient care [4], including simple health problems [3]. In China public hospitals serve the largest number of patients [7], which in consequence leads to long waiting time for various health care services in public hospitals. Such long-time wait also lay heavy caseloads on doctors, which causes minimal individual consulting time. As a result, conflicts between patients and doctors occur due to insufficient communication [4]. It is indicative that the referral mechanism and quality of primary care are in need of improvement in China’s health system.

For the theme of *health outcomes*, patients highlighted treatment effect and recovery length. Although this theme seems to be categorized as a PROM by name, its subthemes are more concerned with PREMs. The subthemes identified are relevant to evaluation of patients’ satisfaction with or experience of treatment effect and recovery length rather than the assessment of patients’ health status or health-related quality of life. These findings imply that the development and application of PROMs, and not only PREMs, could be improved in evaluation of public hospital performance in China.

### Strength of the method

As in China patients’ experiences are rarely considered in assessing hospital care, it is necessary to conduct individual patient interviews as a first step to learn what patients are most concerned with when seeking health services in China’s public hospitals [31]. By using open-ended questions in the interviews, directed initial responses can be avoided and patients are able to convey their views without bias [32, 33]. By using the approach of conventional content analysis, patients’ perception of hospital care could be analyzed without predetermined criteria [30], which could also form the basis for developing an instrument to measure patients’ experiences of hospital care in China.

### Limitations

A limitation of our study is that some patients described the same concepts more than once in the interview. To avoid bias, the research group counted the repeating phrases of the same patient exactly as they appeared in the interview. In future research, attempts can be made to count the repeating concepts of the same respondent only once.

Another limitation is that the only personal information gathered about the patients was their sex, age and residence with some information missing. Since it was an exploratory study with the primary purpose to understand patients’ primary concerns about hospital care, the research group did not set strict rules prior to conducting interviews to gather information on personal data. However, it would be of great value to further explore the difference in perceptions about hospital care between patients of different backgrounds. Patients’ sex, age, education level, employment status and income level might exhibit variation in thematic enunciation which could not be investigated in the current study. This limitation should be addressed and investigated in future studies.

In order to ensure the representativeness of the sample in this study, we used mixed sampling methods to include both outpatients and inpatients from urban and rural areas in Jiangsu Province. However, the results of this current study cannot be generalized to other parts of China, as Jiangsu Province is a relatively prosperous region. But since Jiangsu Province is a health-care reform pilot site, this study could provide an example for similar studies conducted in other parts of the country.

## Conclusion

In conclusion, to the best of our knowledge, this study is the first to explore, identify and prioritize the aspects that patients care most about in China’s public hospitals from patients’ perspectives exclusively. A measurement tool of patient-reported experiences in public hospitals could be built based on our study. The principal concerns of patients about hospital care in Jiangsu Province of China are *environment and facilities*, *professional competence*, *hospital reputation*, *morals of medical staff*, *caring attitudes and emotional support*, *medical costs*, *communication and information*, *efficiency and coordination of care*, and *health outcome*. Efforts should be made to represent patients’ perspectives in PREMs and PROMs to further improve the reform of public hospitals in China.

## Additional files

Additional file 1: The interview guide. The interview guide consisting of six central open-ended questions used in the individual interview was presented in this file. (DOCX 15 kb)

Additional file 2: Themes, subthemes and word frequencies. The themes and subthemes generated through conventional content analysis of transcripts as well as their corresponding word frequencies were presented in this file. (DOCX 21 kb)

## Abbreviations

NCMS: New Rural Cooperative Medical Scheme
PREMs: Patient-Reported Experience Measures
PROMs: Patient-Reported Outcome Measures
UEBMI: Urban Employee Basic Medical Insurance
URBMI: Urban Resident Basic Medical Insurance
