# Supplementary material for: What do patients care most about in China’s public hospitals? Interviews with patients in Jiangsu Province
Source: BMC Health Serv Res. 2018 Feb 8;18:97. doi: 10.1186/s12913-018-2903-6 (PMC5806386; doi:10.1186/s12913-018-2903-6)
Supplement: Supplementary file 1 — The interview guide. The interview guide consisting of six central open-ended questions used in the individual interview was presented in this file. (DOCX 15 kb) [file 12913_2018_2903_MOESM1_ESM.docx]

Additional file 1. The interview guide

1. Please briefly introduce your health status and the past experiences of seeking health care. Could you briefly describe the process and your experience of an impressive clinic visit or hospitalization?

2. What are your principal concerns when you choose a hospital? What are your expectations and requirements for the hospital or the doctor?

3. What are your principal concerns when you choose a doctor? Do you hope that you can be involved in the decision of prescriptions (both medicines and non-medicines) with the doctor? Do you think that the prescribing behavior of most doctors are in line with standards? What are your expectations and requirements for the doctor’s prescribing behavior?

4. Please give evaluations on any part of your hospital visit this time.

5. What is your opinion on the relationship between health professionals and patients? Do you have such experiences yourself that you could talk about?

6. What changes hospitals have made after the initiation of the new health care reform? What changes have taken place in the doctor’s prescribing behavior? Do you have any dissatisfaction with hospitals or doctors currently? What kind of changes you hope hospitals or doctors could make in health care services through the health care reform?
