# Supplementary material for: What do patients care most about in China’s public hospitals? Interviews with patients in Jiangsu Province
Source: BMC Health Serv Res. 2018 Feb 8;18:97. doi: 10.1186/s12913-018-2903-6 (PMC5806386; doi:10.1186/s12913-018-2903-6)
Supplement: Supplementary file 2 — Themes, subthemes and word frequencies. The themes and subthemes generated through conventional content analysis of transcripts as well as their corresponding word frequencies were presented in this file. (DOCX 21 kb) [file 12913_2018_2903_MOESM2_ESM.docx]

Additional file 2. Themes, subthemes and word frequencies

**Table 1. Themes, subthemes and word frequencies under the “structure” category**

| **Themes** | **Word Frequency** | **Subthemes** | **Word Frequency** |
| --- | --- | --- | --- |
| Environment and facilities | 703 | medical equipment | 148 |
|  |  | hospital hygiene | 101 |
|  |  | hospital environment | 74 |
|  |  | close to home | 74 |
|  |  | instruction on the procedure of seeing a doctor | 39 |
|  |  | orderly queue | 38 |
|  |  | number of hospital beds | 33 |
|  |  | transportation to hospital | 31 |
|  |  | infrastructure | 27 |
|  |  | hospital information construction | 26 |
|  |  | hospital canteen | 17 |
|  |  | distribution of clinical departments | 16 |
|  |  | parking | 14 |
|  |  | quiet hospital | 12 |
|  |  | distribution of non-clinical departments | 12 |
|  |  | having a toilet inside the ward | 8 |
|  |  | crowded wards | 7 |
|  |  | supply of hot water | 6 |
|  |  | entertainment | 3 |
|  |  | having an air-conditioner inside the ward | 3 |
|  |  | having a companion bed inside the ward | 3 |
|  |  | companion by logistics service personnel | 3 |
|  |  | having a bathroom inside the ward | 2 |
|  |  | comfortable hospital bed | 2 |
|  |  | providing chairs in the waiting area | 2 |
|  |  | internet surfing inside the ward | 1 |
|  |  | having convenience stores near the hospital | 1 |
| Professional competence | 678 | doctor’s professional capacity | 393 |
|  |  | proper drugs prescribed by the doctor | 73 |
|  |  | doctor’s experience | 48 |
|  |  | ward round of the doctor | 43 |
|  |  | nurse’s professional capacity | 29 |
|  |  | doctor’s reputation | 24 |
|  |  | ward round of the nurse | 24 |
|  |  | doctor’s professional title | 14 |
|  |  | telephone follow-up | 12 |
|  |  | nurse’s experience | 7 |

**Table 1. Themes, subthemes and word frequencies under the “structure” category (continued)**

| **Themes** | **Word Frequency** | **Subthemes** | **Word Frequency** |
| --- | --- | --- | --- |
|  |  | emergency medical services | 5 |
|  |  | doctor’s appearance | 3 |
|  |  | nurse’s appearance | 3 |
| Hospital reputation | 308 | hospital’s scale | 117 |
|  |  | hospital’s medical competence | 92 |
|  |  | hospital’s reputation | 46 |
|  |  | hospital’s rank | 38 |
|  |  | drug supply in the hospital | 15 |
| Morals of medical staff | 221 | appropriate number of drugs prescribed by the doctor | 67 |
|  |  | excessive medical examinations | 58 |
|  |  | personal exchange | 35 |
|  |  | doctor’s medical ethics | 35 |
|  |  | accepting money and gifts by the doctor | 21 |
|  |  | equal care | 5 |

**Table 2. Themes, subthemes and word frequencies under the “process” category**

| **Theme** | **Word Frequency** | **Subtheme** | **Word Frequency** |
| --- | --- | --- | --- |
| Caring attitudes and emotional support | 968 | doctor’s attitude | 181 |
|  |  | attitude | 133 |
|  |  | nurse’s attitude | 107 |
|  |  | doctor’s sense of responsibility | 87 |
|  |  | caring for patients | 48 |
|  |  | doctors thinking of patients | 45 |
|  |  | familiar with doctors | 40 |
|  |  | doctor’s tone | 39 |
|  |  | doctor’s politeness | 37 |
|  |  | trust on the hospital | 37 |
|  |  | doctor’s patience | 34 |
|  |  | doctor’s passion | 31 |
|  |  | satisfying patients’ needs by the doctor | 20 |
|  |  | nurse’s passion | 19 |
|  |  | nurse’s sense of responsibility | 19 |
|  |  | nurse’s tone | 16 |
|  |  | respecting patients’ views | 15 |
|  |  | nurse’s politeness | 8 |
|  |  | consolation from doctors | 8 |
|  |  | satisfying patients’ needs by the nurse | 8 |
|  |  | nurse’s patience | 6 |
|  |  | logistics service personnel’s attitude | 5 |
|  |  | medical technician’s passion | 5 |
|  |  | medical technician’s attitude | 4 |
|  |  | nurses thinking of patients | 3 |
|  |  | caring for the psychological status of patients | 3 |
|  |  | consolation from nurses | 3 |
|  |  | nurses getting on with patients | 3 |
|  |  | doctors getting on with patients | 2 |
|  |  | familiar with the hospital | 1 |
|  |  | privacy protection from the doctor | 1 |
| Medical costs | 541 | medical costs | 231 |
|  |  | health insurance reimbursement | 126 |
|  |  | rate of health insurance reimbursement | 72 |
|  |  | costs of drugs | 54 |
|  |  | scale of health insurance reimbursement | 19 |
|  |  | costs of medical examinations | 11 |
|  |  | costs of registration | 8 |
|  |  | list of items reimbursed by health insurance | 8 |
|  |  | government financial support for serious diseases | 5 |

**Table 2. Themes, subthemes and word frequencies under the “process” category (continued)**

| **Theme** | **Word Frequency** | **Subtheme** | **Word Frequency** |
| --- | --- | --- | --- |
|  |  | real-time reimbursement of health insurance | 4 |
|  |  | costs of surgery | 1 |
|  |  | costs of nursing | 1 |
|  |  | other costs | 1 |
| Communication and information | 482 | communication with the doctor | 91 |
|  |  | discussion on the treatment protocol with the doctor | 60 |
|  |  | doctor’s explanation on patient’s questions | 51 |
|  |  | explanation on drug usage | 45 |
|  |  | explanation on illness | 44 |
|  |  | consultation with the doctor | 42 |
|  |  | time of getting nurses’ responses | 28 |
|  |  | time of getting doctors’ responses | 24 |
|  |  | explanation on the necessity of medical examinations | 23 |
|  |  | communication with the nurse | 14 |
|  |  | explanation on the results of medical examinations | 13 |
|  |  | explanation on illness-related issues | 12 |
|  |  | explanation on the treatment protocol | 9 |
|  |  | doctor’s usage of easy-to-understand language | 9 |
|  |  | explanation on the prognosis | 4 |
|  |  | explanation on medical costs | 3 |
|  |  | explanation on the reimbursement policy | 3 |
|  |  | notice of risks | 3 |
|  |  | explanation on issues pre- and post-surgery | 2 |
|  |  | nurse’s explanation on patient’s questions | 2 |
| Efficiency and coordination of care | 362 | waiting time for diagnosis and treatment | 98 |
|  |  | waiting time for registration | 75 |
|  |  | the procedure of seeing a doctor | 47 |
|  |  | number of patients waiting to see a doctor | 46 |
|  |  | timeliness of obtaining treatment | 32 |
|  |  | waiting time for medical examinations | 27 |
|  |  | waiting time for getting drugs | 14 |
|  |  | the procedure of discharge | 11 |
|  |  | waiting time for paying fees | 8 |
|  |  | waiting time for taking the elevator | 4 |

**Table 3. Themes, subthemes and word frequencies under the “outcome” category**

| **Theme** | **Word Frequency** | **Subtheme** | **Word Frequency** |
| --- | --- | --- | --- |
| Health outcomes | 301 | treatment effect | 253 |
|  |  | time of recovery | 28 |
|  |  | unexpected outcome | 6 |
|  |  | pain control | 5 |
|  |  | disease prognosis | 5 |
|  |  | disease diagnosis | 4 |
